# P059: MRSA surveillance in a Danish region

**DOI:** 10.1186/2047-2994-2-S1-P59

**Published:** 2013-06-20

**Authors:** RA Leth

**Affiliations:** 1 Department of Clinical Microbiology, Aarhus University Hospital, Aarhus, Denmark

## Introduction

Surveillance of MRSA is an important topic in infection control. In our region we have monitored new MRSA cases since 2010.

## Objectives

The aim was to describe surveillance of MRSA in a Danish region with three clinical microbiology departments. The population in the region constitutes approximately 1.2 million inhabitants.

## Methods

Using data from a laboratory information system (MADS) data on new MRSA episodes at each of the three clinical microbiology departments was generated monthly. Data was entered into a common MRSA surveillance database for further follow-up.

## Results

The number of incident MRSA patients has still been increasing since 2010.

In 2012 we registered 225 new MRSA patients in our region, an increase of 24% compared with the number of new MRSA patients in 2011. The number of new cases of livestock MRSA (clonal complex CC398) has also been increasing, from 40 cases in 2011 to 65 cases in 2012.

We did not see any hospital clusters either in 2011 nor in 2012.There were 32 family clusters each including two to five persons.

Totally, more than 62% of the incident patients had an infection with MRSA. Forty-five per cent were exposed by family-members or pigs; in 16% exposure was unknown; 8% were supposedly exposed on holidays outside Europe.

The most common *spa*-types and clonal complex were t034/CC398 (55 cases), t002/CC5 (41 cases), t008/CC8 (12 cases), and t019/CC30 (12 cases).

## Conclusion

The number of MRSA patients is still increasing in the region. In 2012 we did not see any hospital clusters, however, family clusters accounted for 74 patients. Most MRSA patients were exposed by family-members or pigs.

## Disclosure of interest

None declared

